# Screening of Antifungal Lactic Acid Bacteria and Their Impact on the Quality and Shelf Life of Rye Bran Sourdough Bread

**DOI:** 10.3390/foods14071253

**Published:** 2025-04-03

**Authors:** Tianyu Mou, Ruixia Xu, Qin Li, Jianlong Li, Shuliang Liu, Xiaolin Ao, Shujuan Chen, Aiping Liu

**Affiliations:** 1 College of Food Science, Sichuan Agricultural University, Ya’an 625014, China; 2 Key Laboratory of Agricultural Product Processing, Nutrition Health (Co-Construction by Ministry and Province), Ministry of Agriculture and Rural Affairs, Ya’an 625014, China

**Keywords:** antifungal LAB, bread, dough structure, rye bran sourdough

## Abstract

In the present study, *Lactiplantibacillus plantarum* G8, exhibiting higher antifungal activity, and G12, displaying weaker antifungal activity, were isolated from naturally fermented wheat sourdough. Their impacts on bread quality and shelf life were subsequently investigated. The results demonstrated that both strains exhibited robust growth in rye bran sourdough. Compared to the blank control rye bran–wheat flour dough (RB dough), sourdough incorporation enhanced percentages of β-sheet and α-helix secondary structures, facilitating the formation of a more ordered gluten network structure. This contributed to reduced bread baking loss and decreased bread hardness, gumminess, and chewiness, with *Lpb. plantarum* G8 exhibiting the most pronounced effects. Notably, G8 bread displayed superior antifungal efficacy, extending shelf life by 8 d (mold appearance at room temperature: 12 d for G8 vs. 4 d for RB). Furthermore, G8 bread exhibited significantly increased diversity and content of volatile compounds, and received higher preference scores from the sensory panel. This study further advances the development of mold-resistant bakery products.

## 1. Introduction

Rye bran, the seed coat obtained during rye flour milling, is distinguished by its rich mineral, trace element, and phytochemical composition. Moreover, its substantial dietary fiber content has been associated with potential preventive effects against several chronic conditions, including constipation, diabetes, obesity, and coronary heart disease [1]. Consequently, rye bran has attracted increasing interest due to its significant nutritional profile [2].

Bread is a staple food made primarily from wheat flour, yeast, salt, sugar, and water. It is a beloved food across the globe, yet it suffers from a short shelf life and low dietary fiber content. Incorporating rye bran into bread can potentially enhance its dietary fiber content and nutritional value. However, this addition weakens gluten network structure, compromising gas retention capacity, reducing loaf specific volume, and increasing hardness, thereby diminishing the sensory qualities of bread [3]. Furthermore, the high moisture and starch content of bread render it susceptible to fungal contamination, with prevalent genera including *Aspergillus*, *Penicillium*, and *Fusarium*, which can further cause public health problems [4]. Consequently, addressing the detrimental effects of rye bran and preventing bread deterioration remains a critical challenge in food production.

Currently, food spoilage caused by fungi can be effectively controlled by physical or chemical treatments in the industry [5,6]; however, these methods have certain drawbacks, including high costs, energy consumption, nutrient loss, and potential safety risks. Natural preservation methods, such as the use of microorganisms and/or their metabolites, are increasingly applied to prevent spoilage and extend the shelf life of bakery products. These approaches offer high safety and effectiveness, without destroying the characteristics of the bread itself while simultaneously enhancing the nutrition and flavor of the bread. Sourdough, a natural leavening agent produced through the fermentation of cereals, fruits, vegetables, or other substrates with water and microorganisms (primarily *Lactobacillus* and *Saccharomyces cerevisiae*), offers significant potential [7,8,9]. Lactic acid bacteria (LAB)-fermented sourdough not only improves the textural properties of bread, but some also produces microbiologically active compounds that inhibit food spoilage and act as biological preservatives [10,11].

Rye bran is a nutrient-rich and bioactive by-product that serves as a good substrate for LAB fermentation [12]. Dopazo et al. [13] found that *Lpb. plantarum* H1 fermentation with 20% rice bran effectively inhibited fungal growth by producing antifungal metabolites. Moreover, bread supplemented with 20% fermented rice bran extended bread shelf life, without compromising dough and bread quality. To the best of our knowledge, there have been few studies on rye bran bread. This study screened antifungal LAB using four fungal indicator microorganisms; then, the LAB strain with the strongest antifungal activity was utilized to produce rye bran sourdough, and its impact on bread dough characteristics and final product quality was investigated. The findings support the potential development of rye bran sourdough bread with an extended shelf life and improved quality by using antifungal LAB.

## 2. Materials and Methods

### 2.1. Materials and Strains

Wheat flour was purchased from Hebei Jinshahe Flour Industry Group Co., Ltd. (Xingtai, China), while rye bran flour was obtained from Shangnong Grain Shop (Heze, China). The lipid, protein, carbohydrate, moisture, and ash contents of wheat flour were 1.44%, 11.48%, 74.10%, 12.61%, and 0.37%, respectively. Corresponding values for rye bran were 4.22%, 18.61%, 63.28%, 9.75%, and 4.14%. Yanzi high-activity dry yeast was sourced from Guangxi Danbaoli Yeast Co., Ltd. (Laibin, China).

### 2.2. Strains and Growth Conditions

Thirty-four strains, including thirty LAB strains isolated from naturally fermented wheat sourdough (*Lpb. plantarum* LXRR03, LXRR16, G8, LXRR04, LXRR13, LXRR21, LXRR01, G12, and LXRR33; *Leuconostoc citreum* LXRR09, LXRR23, LXRR10, and LXRR34; *Leuconostoc mesenteroides* LXRR29, D3, and D9; *Lactococcus lactis* LXRR12, LXRR24, LXRR15, LXRR02, LXRR05, LXRR11, LXRR31, LXRR35, and LXRR28; *Lactobacillus pantheris* LXRR 26, LXRR30, and LXRR19; *Leuconostoc pseudomesenteroides* LXRR18 and LXRR27) and four fungi (*Aspergillus niger* APN-1, *Penicillium chrysogenum* APF-3, *A. flavus* APE-2, and *A. fumigatus* FUM-1) isolated from wheat and spoiled wheat–rye bran bread, were preserved in the Laboratory of Microbiology, College of Food Science, Sichuan Agricultural University (Ya’an, China). LAB strains were activated in De Man, Rogosa, and Sharpe (MRS) broth at 37 °C for 20 h, while fungi were activated in potato dextrose broth (PDB) at 28 °C for 3~5 d.

### 2.3. Screening of LAB for Their Antifungal Activity

#### 2.3.1. Preliminary Screening

The double-layer method was used for preliminary screening [14]. Briefly, activated LAB strains were inoculated as two parallel lines on MRS agar, with a 2 cm length and 1 cm interval. The plates were incubated at 37 °C for 48 h. PDA semi-solid medium (5 mL) containing 10^5^ spores/mL of each fungus was overlaid. Following complete solidification, plates were incubated at 28 °C for 35~40 h. Strains exhibiting longitudinal inhibition zones greater than 15 mm were selected for further screening.

#### 2.3.2. Further Screening

The microtiter plate-based assay was used to assess antifungal activity as described by Luz et al. [15]. First, cell-free supernatant (CFS) of LAB was prepared by culturing in MRS broth at 37 °C for 48 h, followed by centrifugation (8000× *g*, 10 min, 4 °C) and filtration through a 0.22 μm sterile membrane. In 96-well microtiter plates, 100 μL of CFS was mixed with 100 μL of mold spore suspension in PDB medium (5 × 10^4^ spores/mL). Plates were incubated at 28 °C for 36 h, and absorbance was measured at 560 nm. The antifungal rate was calculated using Equation (1).

(1) $$\mathrm{Antifungal}\;\mathrm{rate}\;\left(\%\right)=100-\frac{{\mathrm{OD}}_{\mathrm{LAB}}-{\mathrm{OD}}_{1}}{{\mathrm{OD}}_{2}-{\mathrm{OD}}_{\mathrm{control}}}\times 100$$

where OD_LAB_ represents the OD_560_ of LAB CFS with inoculated PDB medium, OD_1_ represents the OD_560_ of LAB CFS with uninoculated PDB medium, OD_2_ denotes the OD_560_ of MRS broth with inoculated PDB medium, and OD_control_ denotes the OD_560_ of MRS broth with uninoculated PDB medium.

### 2.4. Preparation of Rye Bran Sourdough and Bread

LAB cells cultured in MRS broth were collected by centrifugation at 5000× *g* for 10 min and subsequently washed twice with sterile physiological saline. The cells were then suspended in purified water and mixed with rye bran to achieve a final concentration of 10^8^ CFU/g and a DY value of 250 (as calculated by Equation (2)). The mixture was incubated at 30 °C for 24 h.

(2) $$\mathrm{DY}=\frac{m_{\mathrm{flour}}+m_{\mathrm{water}}}{m_{\mathrm{flour}}}\times 100$$

where m_flour_ represents the quantity of wheat flour (g), while m_water_ represents the quantity of purified water (g).

Bread was prepared according to the formula outlined in Table 1. Rye bran–wheat flour bread without LAB (RB bread) consisted of 80.0 g wheat flour, 52.0 g water, 6.0 g sugar, 4.0 g butter, 1.0 g salt, 1.5 g yeast, and 8.0 g rye bran. All ingredients, excluding butter, were mixed in a dough mixer, AM-CG108 (Appliance Co. of America, Zhuhai, China), at level 2 speed for 2 min, followed by mixing at level 4 for 5 min. Butter was then added and mixed at level 2 for 2 min. The dough was further mixed at level 4 for 25 min to develop gluten. The dough underwent a double fermentation process, with initial fermentation at 30 °C for 1 h. Afterwards, the dough was rounded and shaped, then fermented again at 30 °C for 1.5 h. The bread was baked in an oven with top and bottom heat temperature at 170 °C for 30 min. G8 and G12 breads were prepared similarly, with the only difference being the substitution of rye bran with rye bran sourdough. *Lpb. plantarum* G8 was selected for rye bran sourdough fermentation due to its higher antifungal activity, while *Lpb. plantarum* G12, which exhibited weaker antifungal activity, served as the negative control. Both strains, belonging to the same species and originating from identical sources, minimized potential interspecies metabolic variations.

### 2.5. Determination of TTA and Lactic and Acetic Acid Contents and Enumeration of LAB in Rye Bran Sourdough

Ten grams of sourdough was homogenized with 90 mL of sterile physiological saline, and the mixture was titrated with 0.1 mol/L NaOH to a pH of 8.50, with the volume of NaOH consumed representing the total titratable acidity (TTA) [16]. Lactic and acetic acid contents were determined by high-performance liquid chromatography (HPLC) following the method of Wu et al. [17]. Rye bran sourdough (10.0 g) was homogenized in 25 mL of 0.01 mol/L H_2_SO_4_, centrifuged at 8000× *g* for 10 min, and filtered through a 0.22 μm membrane. The resulting supernatant was analyzed using an Agilent series 1100 HPLC instrument (Agilent Technologies, Santa Clara, CA, USA) with a Waters Atlantis T3 column (4.6 mm × 250 mm, 5 μm, C18 reverse-phase chromatographic column). The mobile phase consisted of 9 mmol/L H_2_SO_4_, with a flow rate of 0.8 mL/min, column temperature of 30 °C, and detection wavelength of 210 nm. Standard lactic and acetic acid solutions were used for identification and quantification. Enumeration of LAB in the sourdough was performed through plating on an MRS agar plate [18].

### 2.6. Characterization of Rye Bran Bread Dough

The microstructure and gluten proteins of the dough samples were characterized using scanning electron microscopy (SEM) (Hitachi S-4800, Hitachi High-Technologies Corporation, Tokyo, Japan) and Fourier Transform Infrared Spectroscopy (NICOLET iS10, Thermo Fisher Scientific Inc., Madison, WI, USA), respectively, following previous methods [19].

### 2.7. Characterization of Rye Bran Bread

#### 2.7.1. Determination of Texture

The texture of rye bran bread was assessed using the method of Jin et al. with slight modifications [20]. The freshly baked bread was cooled to room temperature and sliced into thin slices measuring 100 mm in length, 100 mm in width, and 12 mm in height. A texture analyzer (TA.XTPlus, Stable Micro System, Godalming, UK) was utilized for the assessment. The measurement parameters included the following: TPA mode, P/36R detection probe, 40% compression degree, pre-test speed of 2 mm/s, test speed of 2 mm/s, post-test speed of 2 mm/s, and trigger force of 5 g.

#### 2.7.2. Determination of Specific Volume and Baking Loss Rate

The specific volume of bread was determined following the method of Fekri et al. [21], while the baking loss rate was assessed according to our previous report [19].

#### 2.7.3. Sensory Evaluation

The sensory evaluation of rye bran bread was conducted with reference to the method by Wu et al. with modifications [22]. A panel of 20 panelists assessed different bread samples, presented in identical plastic containers, with purified water available for mouth rinsing. A 9-point hedonic scale (9: extremely like, 1: extremely dislike) was employed to assess the color, taste, aroma, texture, and overall acceptability of the bread.

#### 2.7.4. Determination of Volatile Flavor Compounds

The volatile flavor compounds in bread samples were analyzed using gas chromatography–mass spectrometry (GC-MS), following a method similar to our previous report [19], with the exception of the GC oven temperature setting. The GC oven temperature was programmed as follows: it was initially held at 50 °C for 2 min, then raised to 220 °C at a rate of 5 °C/min and maintained for 3 min, followed by a further increase to 250 °C at a rate of 3 °C/min. The final temperature was held at 250 °C for 5 min.

### 2.8. Antifungal Effect of LAB in Rye Bran Bread

The freshly baked bread was cooled to room temperature, sliced into thin slices measuring 100 mm in length, 100 mm in width, and 12 mm in height, and maintained in sterile ziplock bags. The samples were kept at room temperature and monitored daily for the presence of fungal colonies on the surface of bread slices [23].

### 2.9. Statistical Analysis

All analyses were performed in triplicate, and the results were presented as mean ± standard deviation. SPSS 27.0 (SPSS, Chicago, IL, USA) was employed for statistical analysis, with one-way analysis of variance (ANOVA) at a 95% confidence interval (*p* < 0.05). Mean comparison was conducted using the least significant difference (LSD) test.

## 3. Results and Discussion

### 3.1. Screening of Antifungal LAB

The antifungal activity of thirty LAB strains was evaluated using a double-layer method with *A. fumigatus*, *A. flavus*, *A. niger*, and *Penicillium flavum* as indicator microorganisms. Ten LAB strains showed inhibitory circle diameters exceeding 15 mm against all four fungi. Then, the antifungal rate of CFS revealed that *Lpb. plantarum* G8 exhibited the strongest antifungal effect, with an inhibition rate ranging from 73.00% to 95.82% (Table 2). Therefore, strain G8 was selected for further investigation of its impact on the quality and shelf life of rye bran sourdough bread. Meanwhile, *Lpb. plantarum* G12, with weaker antifungal activity, was randomly chosen as the control strain. Similarly, in the study by De Simone et al. [24], eight strains of *Lpb. plantarum* with inhibitory activity against *A. niger* were selected. Following 48 h cultivation, all supernatants from the LAB exhibited inhibition rates of fungal radial growth exceeding 10%.

### 3.2. Characterization of Rye Bran Sourdough

Acidification is a key feature of sourdough fermentation, characterized by the production of organic acids, primarily lactic and acetic acids, by LAB. In this study, *Lpb. plantarum* strains with strong (G8) and weaker (G12) antifungal activity were inoculated into rye bran to produce sourdough. After 24 h of fermentation, the TTA in sourdough G12 increased from 3.81 to 23.21 mL, while in sourdough G8, it increased from 3.80 to 32.73 mL, demonstrating significantly higher TTA than sourdough G12 (*p* < 0.05). Lactic and acetic acid contents were 31.97 and 4.24 mg/g in sourdough G8, compared to 15.36 and 1.73 mg/g in sourdough G12. The significant increase in TTA primarily resulted from organic acids produced by LAB metabolism, which contributed to enhanced bread flavor and improved textural characteristics [25].

The viable LAB reached 9.67 log (CFU/g) in sourdough G8 and 9.09 log (CFU/g) in G12, consistent with Boyaci Gunduz et al. [26], indicating robust growth of both strains in rye bran.

### 3.3. Characteristics of Rye Bran Bread Dough

#### 3.3.1. Microstructure of Rye Bran Bread Dough

**Figure 1:**
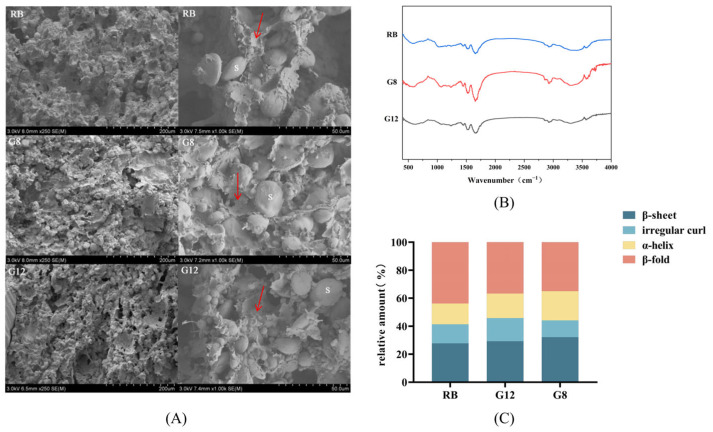
Structure of rye bran bread dough. (**A**) SEM images of bread dough; (**B**) content of dough gluten protein secondary structure; (**C**) FT-IR spectra of dough gluten protein. Note: S: starch granules. The magnification of the left images is 250×, and the magnification of the right images is 1000×; the red arrow points to the gluten network structure.

The bread doughs were freeze-dried and subjected to scanning electron microscopy analysis (Figure 1A). The gluten network structure is the skeletal structure formed by the cross-linking of glutenin subunits through disulfide bonds [27]. Starch granules of various sizes and shapes were present in all bread doughs, encapsulated within the gluten protein network. The addition of rye bran diluted gluten proteins, hindering the formation of the gluten network. As a result, the gluten protein network in RB dough exhibited larger pore sizes, irregular shapes, and a loose structure, with some starch granules exposed. In contrast, bread doughs with rye bran sourdoughs showed a more orderly distribution of starch granules within the gluten protein network, with smaller pore sizes and a dense structure. This was consistent with previous findings by Li et al. [28]. LAB fermentation resulted in the production of organic acids, which in turn increased the activity of proteases in an acidic environment. This led to the accelerated degradation of gluten proteins, strengthening molecular interactions between gluten proteins and wheat starch, and forming a denser gluten network [29]. The enhanced gluten network improved gas retention capacity, reduced pore formation, and resulted in a more uniform, delicate, and soft bread texture [30]. In summary, sourdough incorporation reduced the adverse effects of rye bran on the gluten network.

#### 3.3.2. Secondary Structure of Rye Bran Bread Dough

The amide I band (1600~1700 cm^−1^), derived from the stretching vibrations of the C = O bond, is a key indicator of changes in the secondary structure conformation of gluten proteins. This band is commonly used in studying the secondary structure of gluten proteins [23]. As shown in Figure 1B, β-sheet (1600~1640 cm^–1^) and α-helix (1650~1660 cm^−1^) were the predominant secondary structures in gluten proteins [31]. In comparison to RB dough, the addition of rye bran sourdough led to an increase in the proportion of β-sheet and α-helix, resulting in a more organized gluten network structure. Notably, dough G8 exhibited the highest proportion of β-sheet and α-helix at 53.00%, representing a 6.24% increase over G12, indicating a more ordered gluten network structure in G8 (Figure 1C). This alteration was anticipated to impact the development and quality of the gluten network, endowing the bread with enhanced elasticity and extensibility, aligning with the microstructural results from SEM analysis. Additionally, the irregular curl ranges of the three doughs were similar, ranging from 12.05% to 16.54%, while the proportion of β-fold was significantly different. G8 exhibited the lowest proportion (34.95%), while RB displayed the highest (43.80%).

### 3.4. Characteristics of Rye Bran Bread

#### 3.4.1. Texture of Rye Bran Bread

Studies have shown a negative correlation between bread quality and hardness, gumminess, and chewiness, while resilience is positively correlated [23]. The impacts of antifungal LAB-fermented rye bran sourdough on bread texture are outlined in Table 3. Hardness, a primary texture characteristic, underwent significant changes, with RB exhibiting the highest hardness at 700.97 g, whereas breads containing sourdough (G8, G12) showed reduced hardness by 32.03% and 15.58%, respectively. Additionally, the gumminess and chewiness also decreased in breads G8 and G12, indicating an improvement in bread texture. Similar results were reported by Zhang et al. [32], where bread fermented with sourdough from specific bacterial strains showed decreased hardness after 72 h of storage. Furthermore, the resilience of bread was not significantly affected by the addition of rye bran sourdough (*p* > 0.05). Overall, the incorporation of rye bran sourdough enhanced bread texture, particularly evident in bread G8, resulting in a softer crumb.

#### 3.4.2. Baking Loss Rate and Specific Volume of Rye Bran Bread

As depicted in Table 3, the addition of antifungal LAB-fermented rye bran sourdough reduced the baking loss rate of bread and enhanced its water retention. Bread with rye bran sourdough exhibited a significantly lower baking loss rate compared to RB (*p* < 0.05). Moreover, bread G8 showed the lowest baking loss rate at 6.87%. The inclusion of sourdough improved the gluten network structure, stabilizing gas cells and reducing water loss, thereby lowering the baking loss rate [30,33]. By minimizing water loss, improvements in the textural properties of bread could be achieved, which were consistent with the textural characteristics of the bread.

Compared to RB, the specific volume of bread with antifungal LAB-fermented rye bran sourdough significantly decreased, with a notable difference between breads G8 and G12 (*p* < 0.05). This reduction may be attributed to the high production of organic acids by LAB, leading to a decrease in pH that hindered yeast alcohol fermentation and CO_2_ production [34]. In addition, the degradation of gliadin and glutenin during sourdough fermentation may impact the formation of the gluten protein network and gas retention during bread making [16].

#### 3.4.3. Sensory Evaluation of Rye Bran Bread

**Figure 2:**
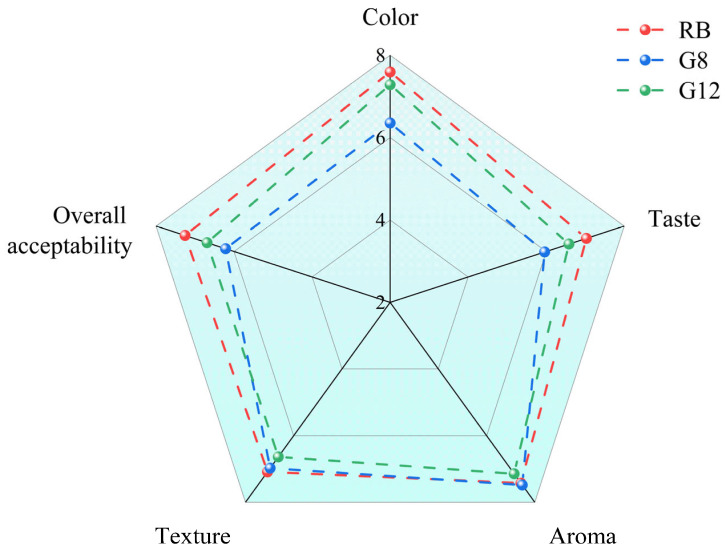
Sensory evaluation of breads.

The sensory evaluation results of various bread samples are shown in Figure 2. Compared to RB, breads with rye bran sourdough received slightly lower sensory scores for taste, color, and overall acceptability, but all scores were above 5.0 points, indicating that the rye bran sourdough bread was acceptable. While RB scored higher for color, the difference was not statistically significant. Meanwhile, sourdough breads scored lower for taste, potentially caused by higher acidity. Texture scores for the bread samples were similar, potentially attributable to the larger specific volume of RB; however, RB exhibited higher hardness. The three types of rye bran breads were considered to have nutty, caramel (characterized by benzaldehyde and 2-pentylfuran), and fermented (yeast) aromatic profiles. Notably, G8 received the highest aroma score, with its odor characterized as more floral and fruitier, potentially correlating with higher content of volatile flavor compounds. Despite G12 not standing out in sensory scores, there were no significant differences compared to RB (*p* < 0.05). Similarly, Eraslan et al. [35] also found that the addition of chickpea sourdough did not negatively impact bread color, aroma, crumb structure, taste, texture, or overall acceptability.

#### 3.4.4. Volatile Flavor Compounds of Rye Bran Bread

The volatile compounds in bread samples were analyzed using GC-MS, revealing a total of 40 volatile compounds from three samples, including 10 aldehydes, 6 alcohols, 3 acids, 13 esters, 4 ketones, and 3 others (Table S1). Compared to RB, the relative content and variety of total volatile compounds in both breads G8 and G12 were significantly higher (*p* < 0.05), particularly in G8 with strong antifungal activity of *Lpb. plantarum*, indicating that the addition of a LAB starter can enhance the aroma profile of bread, providing a rich and diverse sensory experience. Cluster analysis revealed that breads G12 and RB clustered together, indicating certain similarity in their volatile compounds (Figure S1).

Alcohols, key sources of bread aroma with grassy, fruity, or floral aromas, were the highest in G8, being 2.04 and 1.92 times those of RB and G12, respectively. Phenylethyl alcohol, a fragrant compound with a rose-like aroma and found in strawberries, honey, and apples [22], showed the highest abundance in alcohols and positively correlated with bread aroma. The relative contents of phenylethyl alcohol in breads G8 and G12, with added rye bran sourdough, increased compared to RB, being 2.07 and 1.05 times that of RB, respectively. This increase may be attributed to organic acids produced by LAB metabolism, leading to proteolysis of proteins in the flour and release of amino acids serving as precursors to aromatic compounds [36]. 1-Hexanol, an alcohol resulting from fat oxidation in flour [37] and a common volatile flavor component in bread, showed no significant differences in content among the three types of bread (*p* > 0.5).

Esters, known for their low olfactory threshold, are key contributors to the aroma of bread. A total of 13 ester compounds were detected in three types of bread, with relative contents of RB, G8, and G12 at 13.073 μg/kg, 20.991 μg/kg, and 20.857 μg/kg, respectively. The most abundant ester in rye bran sourdough bread was ethyl octanoate, imparting a sweet fruity aroma. Ethyl octanoate was found to be 3.73 times and 3.64 times more prevalent in G12 and G8 compared to RB, respectively. Other esters such as ethyl nonanoate, ethyl hexanoate, ethyl heptanoate, ethyl dodecanoate, ethyl decanoate, 2-phenethyl acetate, and 4-Hydroxynonanoic acid gamma-l also contributed fruity notes, enriching the flavor profile of rye bran bread with a variety of fruit aromas.

The total content of aldehydes in G12 was the highest, with a wide variety of types present. Previous studies have shown that LAB can influence the aldehyde content in sourdough, with benzaldehyde potentially being produced by these bacteria [38]. Benzaldehyde with bitter almond, cherry, and nut aromas was found to be the predominant aldehyde in bread. The relative content of benzaldehyde was the highest in G12, at 4.553 μg/kg, followed by G8 at 3.763 μg/kg, representing 24.13% and 2.59% increases compared to RB, respectively. The content of nonanal in G8 and G12 was significantly lower than that in RB, which may be caused by lipid oxidation. Benzeneacetaldehyde, characterized by its sweet, hawthorn, and honey aroma, exhibited the highest relative content in G12, at 1.978 μg/kg.

Acids not only impart a unique sour taste to bread but also serve as precursors to various flavor substances. The total content of acids in G8 was the highest, measuring 1.59 times that of RB.

Nonanal, benzeneacetaldehyde, benzaldehyde, phenylethyl alcohol, 1-hexanol, ethyl octanoate, ethyl hexanoate, ethyl decanoate, and 2-pentylfuran were the main volatile compounds in rye bran bread. And some compounds were unique to certain breads, thereby contributing distinct flavors. Octanal, hexanal, indole, and 4,6-dimethyl-2,7-nonadiene-5-one were found exclusively in G8, whereas 1-nonanol and hexyl acetate were unique to G12. These differences in volatile compounds were responsible for the distinctive aroma of each bread.

### 3.5. Antifungal Effect of LAB in Rye Bran Sourdough Bread

**Figure 3:**
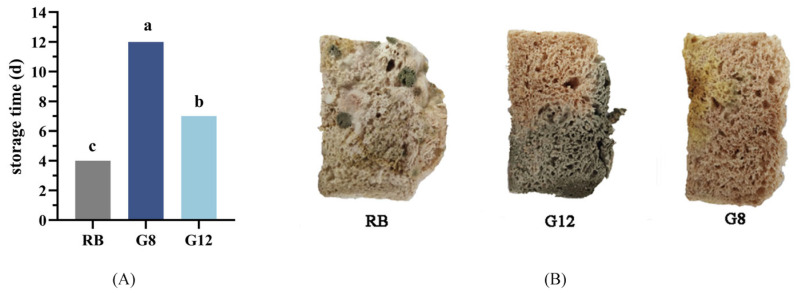
Antifungal effects of LAB in different breads. (**A**) Storage periods of different breads; (**B**) growth of mold on bread slices stored at room temperature for 13 days. Note: Different lowercase letters represent significant differences between each group (*p* < 0.05).

The primary reason for the shortened shelf life of bread is mold contamination. Increasing its shelf life can help minimize both food wastage and potential health risks. The antifungal effect of LAB on mold colonies on bread slices was monitored daily. As shown in Figure 3A, mold growth was observed on RB by the fourth day, whereas bread containing rye bran sourdough showed a longer storage period. Bread inoculated with *Lpb. plantarum* G8 exhibited the longest storage period (*p* < 0.05), reaching 12 d (Figure 3A,B). The extended shelf life exceeded that observed in most previous studies on antifungal LAB-fermented bread [23,39]. The antifungal activity of sourdough LAB in bread may be mainly attributed to the inhibitory metabolites produced during fermentation [40] and synergistic interactions of LAB metabolites with dough or microbe-derived compounds [39,41].

## 4. Conclusions

In this study, *Lpb. plantarum* strains with different antifungal activity were employed to prepare rye bran sourdough. Both LAB strains demonstrated robust growth in rye bran, resulting in increased total titratable acidity. Compared to RB dough, the addition of antifungal LAB-fermented rye bran sourdough promoted the formation of a more organized gluten network structure in bread dough. In addition, it improved bread textural properties, reduced baking loss rate, increased volatile compound diversity and content in bread, and prolonged the antifungal efficiency, particularly with sourdough G8. Despite lower sensory evaluation scores of rye bran sourdough bread compared to RB, G12′s scores did not significantly differ, with G8 still superior in aroma. In conclusion, this study provides a theoretical basis for the development of mold-resistant, shelf-stable bread.

## Figures and Tables

**Table 1:** Bread recipe.

Bread	Wheat Flour (g)	Water (g)	Salt (g)	Yeast (g)	Sugar (g)	Butter (g)	Rye Bran/Rye Bran Sourdough (g)
RB	80.0	52.0	1.0	1.5	6.0	4.0	8.0
G8	80.0	40.0	1.0	1.5	6.0	4.0	20.0
G12	80.0	40.0	1.0	1.5	6.0	4.0	20.0

Note: RB is blank control bread without LAB; G8 is sourdough bread with higher-antifungal-activity LAB; G12 is sourdough bread with weaker-antifungal-activity LAB (negative control).

**Table 2:** Antifungal rates of LAB CFS against different molds.

Strain	Antifungal Rate (%)			
	*A. niger*	*P. chrysogenum*	*A. fumigatus*	*A. flavus*
*Lpb. plantarum* LXRR03	75.64 ± 0.04 ^c^	71.94 ± 0.04 ^cd^	89.13 ± 0.03 ^a^	69.89 ± 0.01 ^c^
*L. Lactis* LXRR12	75.90 ± 0.07 ^c^	82.78 ± 0.04 ^abc^	84.79 ± 0.03 ^abc^	72.22 ± 0.09 ^bc^
*Leu. citreum* LXRR09	95.90 ± 0.03 ^a^	72.22 ± 0.03 ^cd^	86.96 ± 0.00 ^ab^	70.76 ± 0.02 ^bc^
*Lpb. plantarum* LXRR21	92.82 ± 0.01 ^ab^	83.62 ± 0.11 ^abc^	81.52 ± 0.05 ^abc^	71.64 ± 0.00 ^bc^
*Leu. mesenteroides* LXRR29	72.82 ± 0.02 ^c^	63.89 ± 0.08 ^d^	32.61 ± 0.06 ^d^	30.12 ± 0.02 ^d^
*Leu. mesenteroides* D3	82.54 ± 0.00 ^bc^	97.56 ± 0.01 ^a^	79.00 ± 0.03 ^abc^	94.71 ± 0.02 ^a^
*Lpb. plantarum* LXRR04	75.90 ± 0.07 ^c^	87.50 ± 0.07 ^ab^	77.18 ± 0.08 ^bc^	77.49 ± 0.02 ^bc^
*Lpb. plantarum* G8	96.22 ± 0.02 ^a^	95.82 ± 0.04 ^ab^	84.73 ± 0.02 ^abc^	84.75 ± 0.01 ^b^
*Lpb. plantarum* LXRR16	80.77 ± 0.06 ^c^	79.17 ± 0.07 ^bc^	75.00 ± 0.02 ^c^	74.27 ± 0.03 ^bc^
*Lpb. plantarum LXRR13*	83.08 ± 0.01 ^bc^	80.28 ± 0.00 ^bc^	78.26 ± 0.03 ^bc^	78.66 ± 0.01 ^b^

Note: Different lowercase letters indicate significant differences between each column (*p* < 0.05).

**Table 3:** Texture profiles, baking loss rates, and specific volumes of different breads.

Bread	Specific Volume (mL/g)	Baking Loss Rate (%)	Hardness (g)	Chewiness	Resilience	Gumminess
RB	4.32 ± 0.17 ^a^	8.99 ± 0.12 ^a^	700.97 ± 12.34 ^a^	540.74 ± 10.48 ^a^	0.43 ± 0.01 ^a^	572.14 ± 1.61 ^a^
G8	3.33 ± 0.05 ^c^	6.87 ± 0 ^c^	530.91 ± 10.29 ^b^	414.83 ± 25.53 ^c^	0.44 ± 0 ^a^	440.83 ± 10.73 ^c^
G12	3.7 ± 0.08 ^b^	7.84 ± 0.31 ^b^	606.49 ± 38.48 ^b^	473.26 ± 13.36 ^b^	0.43 ± 0.01 ^a^	495.91 ± 23.29 ^b^

Note: Different lowercase letters indicate significant differences between each column (*p* < 0.05).

## Data Availability

The original contributions presented in this study are included in the article/Supplementary Material. Further inquiries can be directed to the corresponding author.

## Supplementary Materials

The following supporting information can be downloaded at https://www.mdpi.com/article/10.3390/foods14071253/s1: Figure S1: Cluster analysis of the volatile compounds in different breads; Table S1: The relative content of volatile flavor compounds in different breads. Different lowercase letters indicate significant differences between each column (*p* < 0.05).
