# Supplementary material for: Screening of Antifungal Lactic Acid Bacteria and Their Impact on the Quality and Shelf Life of Rye Bran Sourdough Bread
Source: Foods. 2025 Apr 3;14(7):1253. doi: 10.3390/foods14071253 (PMC11988746; doi:10.3390/foods14071253)
Supplement: Supplementary file 1 [file foods-14-01253-s001.zip › foods-3549226-Supplementary Table S1.pdf]

Table S1 The relative content of volatile flavor compounds in different breads.

Volatile compounds	Content (μg/kg)		
	RB	G8	G12
**Aldehydes**			
Nonanal	1.652±0.038[a]	0.8±0.266[b]	1.238±0.041[ab]
Benzeneacetaldehyde	1.106±0.022[b]	1.007±0.089[b]	1.978±0.049[a]
(E)-2-Octenal	0.297±0.021[a]	0.292±0.004[a]	0.356±0.037[a]
(E)-2-Nonenal	0.27±0.017[b]	0.39±0.011[a]	0.393±0.056[a]
Decanal	0.152±0.038[a]	0.154±0.059[a]	-
Heptanal	0.136±0.004[a]	0.097±0.004[b]	-
(E)-2-Heptenal	0.123±0.004[b]	-	0.143±0.007[a]
Octanal	-	0.35±0.388[a]	-
Hexanal	-	0.317±0.01[a]	-
Benzaldehyde	3.668±0.369[a]	3.763±0.427[a]	4.553±0.316[a]
**Alcohols**			
Phenylethyl alcohol	35.861±4.501[b]	74.162±5.548[a]	37.582±2.72[b]
1-Hexanol	1.507±0.3[a]	1.561±0.08[a]	1.339±0.161[a]
(Z)-3-Nonen-1-ol	0.176±0.01[a]	-	0.122±0.004[b]
1-Heptanol	0.116±0.018[a]	0.135±0.007[a]	0.148±0.007[a]
1-Octanol	0.077±0.015[b]	0.136±0.003[ab]	0.24±0.066[a]
1-Nonanol	-	-	0.236±0.083[a]
**Esters**			
Ethyl decanoate	4.188±0.434[a]	3.734±0.277[a]	2.757±0.053[b]
Ethyl octanoate	3.66±1.166[b]	13.312±0.1[a]	13.607±0.871[a]
Ethyl hexanoate	1.436±0.125[a]	1.097±0.018[b]	0.937±0.016[b]
2-Phenylethyl acetate	1.37±0.01[a]	-	0.983±0.446[a]
4-Hydroxynonanoic acid gamma-l	0.971±0.054[b]	1.33±0.04[a]	1.008±0.146[b]
Ethyl nonanoate	0.694±0.028[a]	0.836±0.041[a]	0.735±0.077[a]
Ethyl dodecanoate	0.32±0.171[a]	0.207±0.045[a]	0.172±0.098[a]

Ethyl heptanoate	0.234±0.058[a]	0.271±0.013[a]	0.217±0.002[a]
3-Methyl-1-butanol acetate	0.161±0.052[a]	-	0.165±0.055[a]
Pivalic acid, 2-methylpropyl ester	0.039±0.003[a]	-	-
Dichloroacetic acid, 6-ethyl-3-octyl ester	-	0.138±0.024[a]	0.053±0.001[b]
Ethyl 9-decenoate	-	0.066±0.006[a]	0.07±0.021[b]
Hexyl acetate	-	-	0.109±0.001[a]
Acetic acid, trifluoro-, 3,7-dimethyloctyl ester	-	-	0.044±0.008[a]
**Acids**			
Octanoic acid	0.248±0.058[a]	-	-
Isovaleric acid	-	0.263±0.028[a]	-
2-Methyl butyric acid	-	0.131±0.012[a]	0.079±0.041[a]
**Ketones**			
2-Nonanone	0.203±0.014[b]	0.265±0.022[a]	0.26±0.007[a]
2,4,6-Tris(1,1-dimethylethyl)-4-methylcyclohexa-2,5-dien-1-one	-	0.234±0.019[a]	0.13±0.07[b]
2,5-Hexanedione	-	0.174±0.082[a]	-
4,6-Dimethyl-2,7-nonadien-5-one	-	0.137±0.012[a]	-
**Others**			
2-Pentylfuran	1.663±0.027[a]	1.94±0.223[a]	2.006±0.233[a]
Indole	-	1.812±0.033[a]	-
5-Amino-1-methyl-1H-pyrazole-4-carboxamide	-	-	0.171±0.013[a]

Note: "-", not found; different lowercase letters indicate significant differences between each column ($P < 0.05$).
