# Supplementary figures and images for: Screening of Antifungal Lactic Acid Bacteria and Their Impact on the Quality and Shelf Life of Rye Bran Sourdough Bread
Source: Foods. 2025 Apr 3;14(7):1253. doi: 10.3390/foods14071253 (PMC11988746; doi:10.3390/foods14071253)

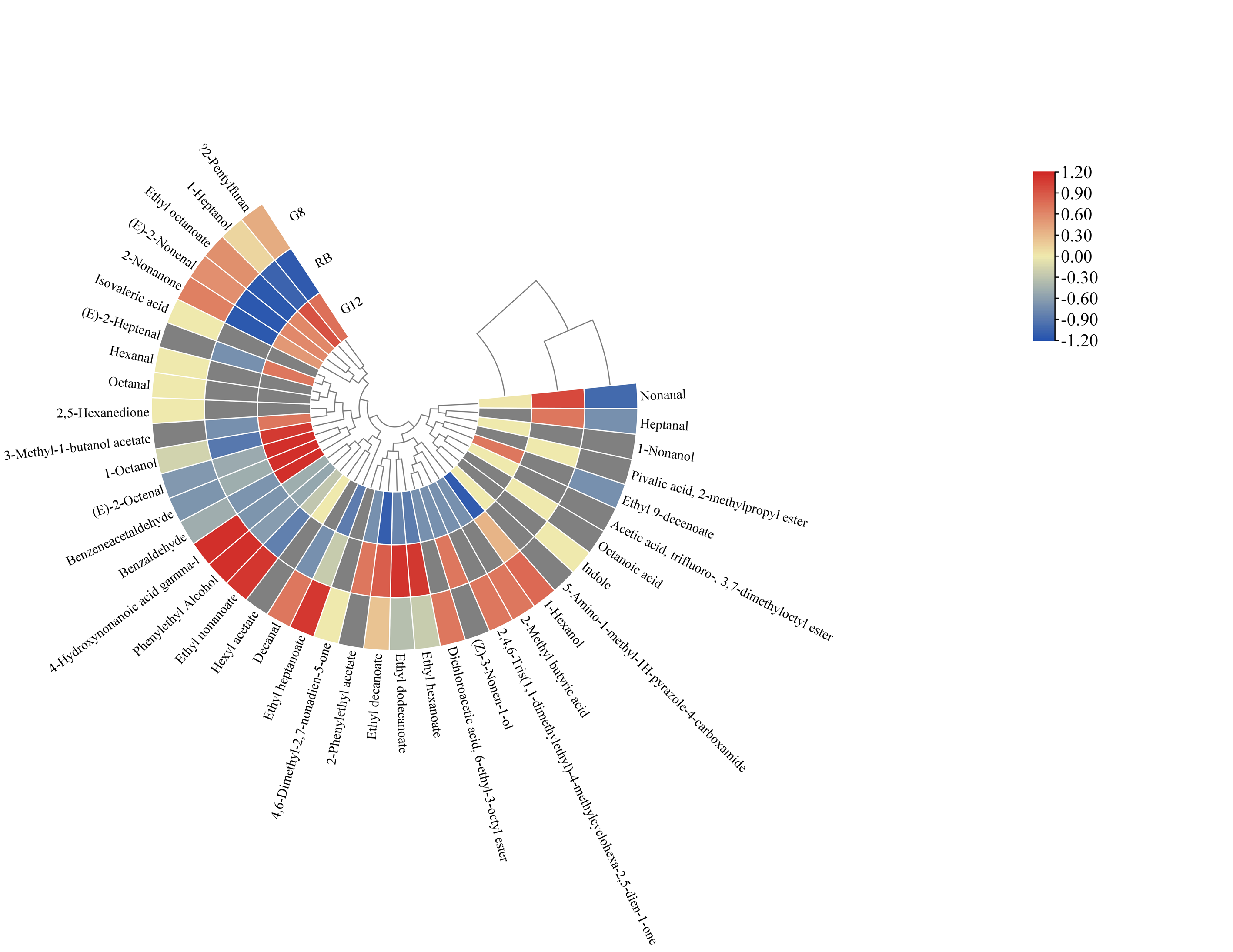

Supplement: Supplementary file 1 [file foods-14-01253-s001.zip › foods-3549226-Supplementary Figure S1.tif]
